# Percentage of Deaths Associated With Inadequate Physical Activity in the United States

**DOI:** 10.5888/pcd18.170354

**Published:** 2018-03-29

**Authors:** Susan A. Carlson, E. Kathleen Adams, Zhou Yang, Janet E. Fulton

**Affiliations:** 1 National Center for Chronic Disease Prevention and Health Promotion, Centers for Disease Control and Prevention, Atlanta, Georgia; 2 Department of Health Policy and Management, Rollins School of Public Health, Emory University, Atlanta, Georgia

## Abstract

**Introduction:**

Current physical activity guidelines recommend that adults participate weekly in at least 150 minutes of moderate-intensity equivalent aerobic physical activity to achieve substantial health benefits. We used a nationally representative sample of data of US adults to estimate the percentage of deaths attributable to levels of physical activity that were inadequate to meet the aerobic guideline.

**Methods:**

Data from the 1990 to 1991 National Health Interview Survey for adults aged 25 years or older were linked with mortality data up until December 31, 2011, from the National Death Index (N = 67,762 persons and 18,796 deaths). Results from fully adjusted Cox proportional hazards models were used to estimate hazard ratios and population attributable fractions for inadequate levels of physical activity (ie, less than 150 minutes per week of moderate-intensity equivalent aerobic activity).

**Results:**

Overall, 8.3% (95% confidence interval [CI], 6.4–10.2) of deaths were attributed to inadequate levels of physical activity. The percentage of deaths attributed to inadequate levels was not significant for adults aged 25 to 39 years (−0.2%; 95% CI, −8.8% to 7.7%) but was significant for adults aged 40 to 69 years (9.9%; 95% CI, 7.2%–12.6%) and adults aged 70 years or older (7.8%; 95% CI, 4.9%–10.7%).

**Conclusions:**

A significant portion of deaths was attributed to inadequate levels of physical activity. Increasing adults’ physical activity levels to meet current guidelines is likely one way to reduce the risk of premature death in the United States.

## Introduction

Regular participation in physical activity reduces the risk of dying prematurely (1). The 2008 US Department of Health and Human Services Physical Activity Guidelines for Americans recommend that adults participate in at least 150 minutes of moderate intensity-equivalent aerobic activity weekly for substantial health benefits, and for additional and more extensive health benefits adults should increase this to more than 300 minutes per week (2). Population levels of physical activity inadequate to meet guidelines can place a burden on the US population in terms of premature mortality. 

The population attributable fraction (PAF) (3) provides an estimate of the percentage of deaths that could be averted if people who do not meet the guideline were active at levels consistent with the guideline. Studies have estimated the PAF for physical inactivity by using an equation that combines estimates of risk from one source and prevalence from another source (4–7). Combining estimates across sources can bias results if the characteristics of the 2 source populations differ, if the measure of physical activity differs across sources, or if confounding is not accounted for (3,8). Estimating the PAF from a survival analysis conducted in a single population can help overcome these biases. No study has estimated the PAF for inadequate levels of physical activity (using criteria based on guidelines) (2) from a survival analysis of a nationally representative sample.

Evidence of the association between physical activity and mortality comes from studies focused on middle-aged adults and mainly includes adults aged 40 years or older, with few data available for adults aged 80 years or older (1,7). Researchers who examined the influence of age on the association between physical activity and mortality suggested that the association generally gets stronger with increasing age (9–11). However, one study with 42 years of follow-up found that the PAFs for physical inactivity were consistent across age groups, ranging from 7.3% (aged 20–44 y) to 9.1% (aged ≥65 y) (11).

Our study had 2 objectives. The first was to examine the influence that physical activity level (defined using the 2008 Physical Activity Guidelines for Americans aerobic criteria) has on mortality in a nationally representative sample of the US population and to examine this association by age group. The second was to estimate the proportion of deaths attributable to inadequate levels of physical activity in US adults.

## Methods

### Data

We analyzed data from the 1990 and 1991 National Health Interview Survey (NHIS), which were linked to the National Death Index (NDI) (12,13). Vital status information was available from January 1, 1990, through December 31, 2011.

The NHIS collects basic health and demographic information on all household members; additional information, such as that for physical activity, is collected on one randomly selected adult through a face-to-face survey (12). NHIS interviews were completed by 84,836 (1990, n = 41,104; 1991, n = 43,732) adult respondents. The response rates for the adult supplements were 83.4% in 1990 and 87.8% in 1991 (12). Of the 84,836 respondents, 83,971 (99%) were matched with NDI records and had known vital status information (number of decedents = 22,305) (13).

In 1990 and 1991, there were 74,346 adult respondents of the NHIS sample aged 25 years or older with a matched NDI record (22,101 decedents). First, 24 adults (12 decedents) with missing data on date of birth were excluded. Second, we excluded 3,685 adults (2,338 decedents) who were physically disabled or whose disability status was unknown and, consequently, were not asked all physical activity questions. Third, we excluded 2,838 adults (918 decedents) who had missing physical activity or covariate data. Finally, we excluded 37 adults who died in the same quarter in which they completed the NHIS interview (final sample, n = 67,762; decedents, 18,796).

### Measures

**Physical activity assessment. **In the 1990 and 1991 surveys, participants were asked if they had engaged in any exercises, sports, or physically active hobbies in the past 2 weeks (14). If they responded yes, they were asked how often they did each activity during the preceding 2 weeks and the average number of minutes they participated each time. We included participation in 13 physical activities (ie, walking; jogging or running; gardening or yard work; aerobics or aerobic dancing; tennis; biking; swimming; basketball; baseball or softball; football; soccer; volleyball; and handball, racquetball, or squash) (14).

We categorized activities as moderate-intensity or vigorous-intensity for each person. This was done by first estimating a 60% maximal oxygen uptake (VO_2max_) value for each adult on the basis of standard equations that account for sex and age (15). An adult’s estimated 60% VO_2max_ was compared with an assigned Metabolic Equivalent of Task (MET) value (16). If the MET value of the activity was higher than the adult’s estimated 60% VO_2max_, then the activity was categorized as vigorous intensity; otherwise the activity was categorized as moderate intensity.

A minute of vigorous-intensity activity was counted as 2 minutes of moderate-intensity activity to calculate minutes of moderate-intensity equivalent activity (2). Using minutes of moderate-intensity equivalent activity and guidelines criteria, adults were categorized into a 4-level physical activity variable: inactive (no physical activity reported in the preceding 2 weeks), insufficiently active (some activity but <150 min/wk), sufficiently active (150–300 min/wk), and highly active (>300 min/wk). Adults were then categorized into a 3-level variable: inactive, insufficiently active, and active (combining sufficiently and highly active [ie, ≥150 min/wk]) (2).

**Time scales.** The public-use data set provided the month and year of birth, quarter and year of the interview, and quarter and year of death. To create the time scale, the fifteenth day of the birth month was used along with the midpoint for the interview and midpoint of the death quarter.

**Covariates.** In the NHIS, interviewers assessed sex, race/ethnicity, education, cigarette smoking, and hypertension (12). Categorical variables were created for each of these covariates: sex (male, female), race/ethnicity (non-Hispanic white, non-Hispanic black, other), education level (less than high school graduate, high school graduate, some college, college graduate), smoking status (never smoker, former smoker, current smoker), and hypertension (yes, no). Body mass index (BMI, calculated as reported weight in kilograms divided by the square of reported height in meters [kg/m^2^]) was categorized as underweight (<18.5), normal weight (18.5–24.9), overweight (25.0–29.9), or obese (≥30) (17).

### Statistical analysis

We compared the prevalence of physical activity levels for adults who died with those for adults who survived to the end of follow-up by using adjusted Wald tests; significance was set at *P* < .05. We used Cox proportional hazards models to estimate hazard ratios (HRs) and 95% confidence intervals (CIs) by level of physical activity, while adjusting for sex, race/ethnicity, education level, smoking status, hypertension, and BMI category. We used sensitivity analyses to examine all estimates after removing adults who died during the first 2 years of follow-up.

We made an a priori decision to examine the association between physical activity and mortality by age, and testing the interaction between age and physical activity level supported this decision. We began with 3 age groups (25–39 y, 40–79 y, and ≥80 y) because previous research has focused on adults aged 40 years or older, with minimal data for those aged 80 years or older (1,7). We decided to use a cut-off of 70 years or older for the highest age group, because we found the association of activity with mortality for those aged 70 to 79 was more similar to adults in the oldest age group (≥80 y) than to those aged 40 to 69. Our final age groups were 25 to 39 years, 40 to 69 years, and 70 years or older. Age was used as the time scale in the Cox models, with age at death or the end of follow-up (December 31, 2011) as the survival time and age at entry into the study as left-censoring (18). The Breslow method was used for handling tied failure times (19).

All analyses were conducted using STATA (StataCorp LP) version 13. PAFs and their corresponding 95% CIs were calculated directly from the results of the fully adjusted Cox models (20). HRs were not significantly different for the sufficiently and highly active levels for any age group; therefore, the 3-level physical activity variable was used to estimate PAFs. We applied survey weights and adjusted for the complex sample design of the NHIS (12).

## Results

Overall, the prevalence of physical inactivity was significantly higher among adults who died than those who survived through follow-up, while prevalence of both insufficient and sufficient activity was significantly higher among those who survived than those who died (Table 1). The same significant differences were observed in adults aged 25 to 39 years and 40 to 69 years (Figure). In adults aged 70 or older, prevalence of inactivity was significantly higher, and prevalence of being highly active was lower, among those who died than among those who survived.

**Table 1:** Select Characteristics of Study Participants, Decedents, and Survivors, National Health Interview Survey Linked Mortality Files, 1990–1991

Characteristic	Study Participants^a^		Among Decedents		Among Survivors	
	No.	% (SE)	No.	% (SE)	No.	% (SE)
**Total**	67,762	100 (NA)	18,796	100 (NA)	48,966	100 (NA)
**Physical activity level^b^**						
Inactive	23,623	34.7 (0.5)	7,857	41.2 (0.7)	15,766	32.6 (0.5)
Insufficiently active	17,351	25.8 (0.2)	3,988	21.4 (0.4)	13,363	27.3 (0.3)
Sufficiently active	11,736	17.4 (0.2)	2,775	14.7 (0.3)	8,961	18.2 (0.2)
Highly active	15,052	22.1 (0.3)	4,176	22.8 (0.5)	10,876	21.9 (0.4)
**Sex**						
Male	28,444	47.6 (0.2)	8,227	50.2 (0.4)	20,217	46.7 (0.3)
Female	39,318	52.4 (0.2)	10,569	49.8 (0.4)	28,749	53.3 (0.3)
**Age, y**						
25–39	27,383	40.8 (0.3)	1,365	7.7 (0.3)	26,018	52.0 (0.3)
40–69	30,929	47.7 (0.3)	9,142	52.4 (0.5)	21,787	46.1 (0.3)
≥70	9,450	11.6 (0.2)	8,289	39.9 (0.5)	1,161	1.9 (0.1)
**Race/ethnicity**						
White, non-Hispanic	52,791	79.3 (0.5)	15,379	83.9 (0.5)	37,412	77.7 (0.5)
Black, non-Hispanic	8,558	10.1 (0.4)	2,373	9.7 (0.4)	6,185	10.2 (0.4)
Other	6,413	10.7 (0.4)	1,044	6.4 (0.4)	5,369	12.1 (0.4)
**Education**						
Less than HS graduate	14,209	20.0 (0.3)	6,970	35.4 (0.6)	7,239	14.7 (0.3)
HS graduate	25,198	37.7 (0.3)	6,672	36.0 (0.4)	18,526	38.3 (0.4)
Some college	13,335	19.7 (0.2)	2,734	14.9 (0.3)	10,601	21.4 (0.2)
College graduate	15,020	22.6 (0.3)	2,420	13.6 (0.4)	12,600	25.6 (0.4)
**Smoking status**						
Never	32,395	47.2 (0.3)	7,801	39.3 (0.5)	24,594	49.9 (0.3)
Former	17,304	26.5 (0.3)	5,908	33.0 (0.4)	11,396	24.2 (0.3)
Current	18,063	26.3 (0.2)	5,087	27.7 (0.5)	12,976	25.9 (0.2)
**Hypertension**						
Yes	16,554	23.3 (0.2)	8,222	42.4 (0.5)	8,332	16.9 (0.2)
No	51,208	76.7 (0.2)	10,574	57.6 (0.5)	40,634	83.1 (0.2)
**Body mass index**						
Underweight	1,966	2.6 (0.1)	696	3.3 (0.1)	1,270	2.4 (0.1)
Normal weight	33,661	48.9 (0.2)	8,392	44.0 (0.4)	25,269	50.6 (0.3)
Overweight	22,366	34.1 (0.2)	6,600	36.2 (0.4)	15,766	33.4 (0.3)
Obese	9,769	14.3 (0.2)	3,108	16.5 (0.3)	6,661	13.6 (0.2)
**Baseline year**						
1990	33,475	50.7 (0.2)	9,600	52.5 (0.4)	23,875	50.1 (0.2)
1991	34,287	49.3 (0.2)	9,196	47.5 (0.4)	25,091	49.9 (0.2)

Abbreviations: HS, high school; NA, not applicable; SE, standard error.

a The original sample size of adults aged 25 years or older was 74,346. First, 24 adults missing month or year of birth were excluded. Second, adults categorized as physically disabled or whose physical disability status was unknown were excluded (n = 3,685) because they were not asked all physical activity questions. Third, adults missing data on physical activity (n = 1,113), covariates (n = 1,617), or both (n = 108) were excluded. Finally, 37 adults who died the same quarter of the year as interviewed were excluded.

b Individuals were categorized into 4 activity levels based on guidelines: inactive (no physical activity reported in the past 2 weeks), insufficiently active (some activity but less than 150 min/wk of moderate-intensity equivalent activity), sufficiently active (150–300 min/wk of moderate-intensity equivalent activity), and highly active (>300 min/wk of moderate-intensity equivalent activity).

**Figure:**
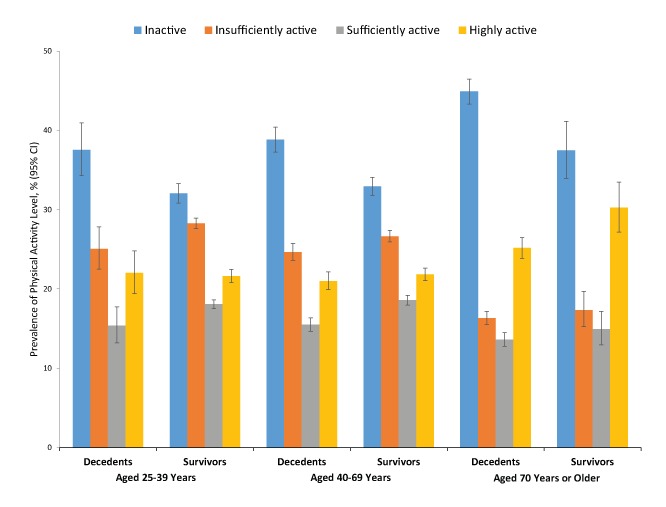
Prevalence of physical activity among decedents and survivors aged 25 years or older (N = 67,762), by age group, National Health Interview Survey Linked Mortality Files, 1990–1991. Adults who were excluded from the sample were those 1) categorized as physically disabled or whose disability status was unknown; 2) missing mortality or time scale data; missing data on physical activity, covariates, or both; and who died the same quarter of the year as interviewed. Individuals were categorized into 4 activity levels: inactive (no physical activity reported in the past 2 weeks), insufficiently active (some activity but <150 min/wk of moderate-intensity equivalent activity), sufficiently active (150–300 min/wk of moderate-intensity equivalent activity), and highly active (>300 min/wk of moderate-intensity equivalent activity). Abbreviation: CI, confidence interval. [A tabular description of this figure is available.] **Table:** 

Age Group	Decedents	Survivors
	Prevalence of Physical Activity Group, % (95% CI)	
**25–39 y**		
Inactive	37.6 (34.3–41.0)	32.0 (30.8–33.3)
Insufficiently active	25.1 (22.5–27.8)	28.3 (27.6–28.9)
Sufficiently active	15.4 (13.2–17.8)	18.1 (17.5–18.6)
Highly active	22.0 (19.4–24.8)	21.6 (20.8–22.4)
**40–69 y**		
Inactive	38.8 (37.2–40.4)	32.9 (31.8–34.1)
Insufficiently active	24.7 (23.6–25.8)	26.6 (25.9–27.4)
Sufficiently active	15.5 (14.7–16.4)	18.6 (18.0–19.2)
Highly active	21.0 (19.9–22.2)	21.8 (21.1–22.6)
**≥70 y**		
Inactive	44.9 (43.4–46.4)	37.5 (34.0–41.1)
Insufficiently active	16.3 (15.5–17.2)	17.4 (15.2–19.7)
Sufficiently active	13.6 (12.8–14.5)	14.9 (12.9–17.2)
Highly active	25.2 (23.9–26.5)	30.2 (27.2–33.5)

When physical activity was categorized into 4 levels and models adjusted for covariates, physical activity was significantly associated with mortality for adults aged 40 to 69 (adjusted Wald *P* <.001) and 70 or older (*P* <.001), while the association was not significant for adults aged 25 to 39 at baseline (*P* = .48) (Table 2). Among adults aged 40 to 69 and 70 or older, inactive adults had an increased risk of premature death compared with sufficiently active adults. For adults aged 40 to 69, the difference was also significant for those insufficiently active versus sufficiently active. For adults aged 25 to 39, inactive adults compared with those sufficiently active had an increased risk of premature death in the unadjusted models; however, once we controlled models for demographic characteristics, this increased risk was no longer significant. No significant differences between those who were highly active versus sufficiently active were observed for any age group. All findings were similar if adults who died in the first 2 years of follow-up were excluded, except while physical activity level remained a significant variable in the model for those 70 or older (*P* = .001), the comparison between the inactive group and sufficiently active group was no longer significant (*P* = .13).

**Table 2:** Hazard Ratios for All-Cause Mortality, by Age Group and Physical Activity Level, National Health Interview Survey Linked Mortality Files, 1990–1991

Age Group and Physical Activity Level^a^	Overall^b^		Fully Adjusted^c^ HR (95% CI), Excluding Adults Who Died During First 2 Years of Follow-Up^d^
	Unadjusted HR (95% CI)	Fully Adjusted^c^ HR (95% CI)	
**4-Level Physical Activity Variable**			
**25–39 y**			
Inactive	1.38 (1.13–1.69)	1.10 (0.90–1.35)	1.09 (0.88–1.34)
Insufficiently active	1.05 (0.86–1.27)	0.99 (0.81–1.21)	1.02 (0.83–1.24)
Sufficiently active	1.00 [Reference]	1.00 [Reference]	1.00 [Reference]
Highly active	1.18 (0.95–1.46)	1.11 (0.89–1.37)	1.12 (0.90–1.39)
*P* value^e^	.003	.48	.63
**40–69 y**			
Inactive	1.42 (1.34–1.51)	1.28 (1.20–1.36)	1.26 (1.18–1.34)
Insufficiently active	1.15 (1.07–1.24)	1.14 (1.05–1.23)	1.13 (1.05–1.22)
Sufficiently active	1.00 [Reference]	1.00 [Reference]	1.00 [Reference]
Highly active	1.08 (1.00–1.16)	1.05 (0.98–1.14)	1.06 (0.98–1.14)
*P* value^e^	<.001	<.001	<.001
**≥70 y**			
Inactive	1.10 (1.03–1.18)	1.11 (1.03–1.19)	1.06 (0.98–1.14)
Insufficiently active	1.04 (0.96–1.13)	1.07 (0.98–1.16)	1.06 (0.97–1.15)
Sufficiently active	1.00 [Reference]	1.00 [Reference]	1.00 [Reference]
Highly active	0.96 (0.89–1.04)	0.93 (0.86–1.01)	0.93 (0.86–1.01)
*P* value^e^	<.001	<.001	.001
**3-Level Physical Activity Variable**			
**25–39 y**			
Inactive	1.26 (1.08–1.47)	1.04 (0.89–1.21)	1.02 (0.87–1.19)
Insufficiently active	0.95 (0.82–1.11)	0.94 (0.81–1.09)	0.95 (0.82–1.11)
Active	1.00 [Reference]	1.00 [Reference]	1.00 [Reference]
*P* value^e^	.002	.45	.70
**40–69 y**			
Inactive	1.36 (1.29–1.44)	1.24 (1.18–1.31)	1.22 (1.16–1.28)
Insufficiently active	1.10 (1.04–1.17)	1.11 (1.04–1.17)	1.10 (1.03–1.17)
Active	1.00 [Reference]	1.00 [Reference]	1.00 [Reference]
*P* value^e^	<.001	<.001	<.001
**≥70 y**			
Inactive	1.13 (1.07–1.19)	1.16 (1.09–1.23)	1.11 (1.05–1.17)
Insufficiently active	1.07 (1.00–1.15)	1.12 (1.04–1.20)	1.11 (1.03–1.19)
Active	1.00 [Reference]	1.00 [Reference]	1.00 [Reference]
*P* value^e^	<.001	<.001	<.001

Abbreviations: CI, confidence interval; HR, hazard ratio.

a Individuals were categorized into 4 activity levels on the basis of guidelines: inactive (no physical activity reported in the past 2 weeks), insufficiently active (some activity but less than 150 min/wk of moderate-intensity equivalent activity), sufficiently active (150–300 min/wk of moderate-intensity equivalent activity), and highly active (>300 min/wk of moderate-intensity equivalent activity). When categorized into 3 levels, adults sufficiently active and highly active were combined (ie, active).

b Includes 67,762 adults aged 25 years or older. Adults who were excluded from the sample were adults categorized as physically disabled or whose physical disability status was unknown; adults missing mortality or time scale data; adults missing data on physical activity, covariates, or both; and adults who died the same quarter of the year as interviewed.

c Covariates were sex, race/ethnicity, education, smoking status, hypertension, and body mass index category.

d Excluded 1,040 adults (aged 25–39 y, n = 47; aged 40–69 y, n = 381; aged ≥70 y, n = 612).

e *P* values determined by using adjusted Wald test for the joint significance of the levels of the given physical activity variable.

When physical activity level was categorized into 3 levels and the comparison group was active adults, findings were similar (Table 2). Results were similar for adults aged 40 to 69 and 70 or older, with both inactive and insufficiently active adults versus active adults having an increased risk of premature death. There was no significant association (*P* = .45) between the 3-level physical activity variable and mortality for adults aged 25 to 39. Findings were similar if those who died in the first 2 years of follow-up were excluded.

Overall, 8.3% (95% confidence interval [CI], 6.4%–10.2%) of deaths were attributed to inactive and insufficiently active levels of physical activity (ie, levels of physical activity inadequate to meet the minimal guideline) (Table 3). For adults aged 40 to 69, 9.9% of deaths were attributed to inadequate levels of physical activity, and for adults aged 70 or older, 7.8% of deaths were attributed to inadequate levels of physical activity. The percentage of deaths attributed to inadequate levels of physical activity was not significantly different for adults aged 25 to 39. PAFs decreased after removal of adults who died in the first 2 years, but all significant findings remained significant.

**Table 3:** Population Attributable Fractions for All-Cause Mortality, by Age Group and Physical Activity Level, National Health Interview Survey Linked Mortality Files, 1990–1991

Age Group and Physical Activity Level^a^	PAF^b^ Overall^c^	PAF^b^ Excluding Adults Who Died in <2 y^d^
	% (95% CI)	
**Overall**		
Inactive	6.5 (5.2 to 7.9)	5.4 (4.0 to 6.7)
Insufficiently active	1.8 (0.9 to 2.7)	1.7 (0.8 to 2.6)
Inactive and insufficiently active	8.3 (6.4 to 10.2)	7.0 (5.1 to 8.9)
**25–39 y**		
Inactive	1.4 (−4.5 to 7.0)	0.6 (−5.2 to 6.2)
Insufficiently active	−1.7 (−5.6 to 2.2)	−1.3 (−5.4 to 2.6)
Inactive and insufficiently active	−0.2 (−8.8 to 7.7)	−0.7 (−9.3 to 7.3)
**40–69 y**		
Inactive	7.6 (5.7 to 9.4)	6.9 (5.0 to 8.7)
Insufficiently active	2.4 (0.9 to 3.8)	2.2 (0.7 to 3.6)
Inactive and insufficiently active	9.9 (7.2 to 12.6)	9.1 (6.3 to 11.7)
**≥70 y**		
Inactive	6.1 (3.9 to 8.3)	4.2 (2.0 to 6.4)
Insufficiently active	1.7 (0.6 to 2.8)	1.6 (0.5 to 2.7)
Inactive and insufficiently active	7.8 (4.9 to 10.7)	5.8 (2.9 to 8.6)

Abbreviations: CI, confidence interval; PAF; population attributable fraction.

a Individuals were categorized into 3 activity levels based on guidelines: inactive (no physical activity reported in the past 2 weeks), insufficiently active (some activity but less than 150 min/wk of moderate-intensity equivalent activity), and active (greater than or equal to 150 min/wk of moderate-intensity equivalent activity).

b PAFs are estimated directly from adjusted Cox proportional hazards models that included: sex, race/ethnicity, education, smoking status, hypertension, and BMI category. To calculate the overall PAF, models were estimated using the full population with each age group having a separate baseline hazards and interacted with each variable included.

c Includes 67,762 adults aged 25 years or older. Adults who were excluded from the sample were adults categorized as physically disabled or whose physical disability status was unknown; adults missing mortality or time scale data; adults missing data on physical activity, covariates, or both; and adults who died the same quarter of the year as interviewed.

d Excluded 1,040 adults (aged 25–39 y, n = 47; aged 40–69 y, n = 381; aged ≥70 y, n = 612).

## Discussion

In a nationally representative sample of nondisabled US adults, inadequate levels of physical activity were associated with an increased risk of premature death. Overall, 8.3% of deaths in nondisabled adults 25 or older were attributed to inadequate levels of physical activity. The percentage of deaths attributed to inadequate physical activity was significant for adults aged 40 to 69 (9.9%) and among adults aged 70 or older (7.8%). On the basis of these findings, increasing adults’ physical activity to levels consistent with current guidelines and *Healthy People 2020* objectives may decrease the risk of premature death in the United States (2,21).

When comparing our results to those of studies that examined the association between higher levels of physical activity and overall mortality, we found that our estimates were similar or lower (10,22–24). For example, in a meta-analysis when studies using 3 levels of physical activity were summarized, the combined relative risk for the most active group was 0.78 (22). Taking the inverse would give an estimate of 1.28, which is very close to our estimate of 1.24 for adults aged 40 to 69, and a little higher than our estimate of 1.19 for adults aged 70 or older. A pooled analysis of 6 studies from the National Cancer Institute Cohort Consortium (median entry age, 62 y; range, 21–98 y; median follow-up, 14.2 y) reported a hazard ratio of 0.80 (inverse, 1.25) for those performing less than the guideline recommends compared with those who were inactive; 0.69 (inverse, 1.45) for those performing 1 to 2 times the recommended amount; and 0.63 (inverse, 1.59) for those performing 3 to 5 times the recommended amount (23).

Comparing our PAF estimates with those of other studies is difficult because of the various methods and measures of physical activity used. One study that combined physical activity prevalence estimates from surveillance systems with risk estimates from the literature reported a PAF for mortality relative to physical inactivity (defined as an activity level insufficient to meet recommendations) in the United States of 10.8% (95% CI, 8.6%–13.1%) (7). This estimate may be higher, because the estimates of risks came mainly from studies of physical activity and all-cause mortality in people aged 40 years or older, with limited data for those aged 80 or older (7). This may be the reason that it is closer to our estimate of 9.9% for those aged 40 to 69 years. Another study using data from the National Health and Nutrition Examination Survey that were linked with mortality data attributed 10.9% (95% CI, 3.0%–18.7%) of deaths to non-ideal physical activity (ideal is defined as participation in moderate-intensity [≥5 times/wk] or vigorous-intensity [≥3 times/wk] aerobic physical activity); however, these levels are not equivalent to current guidelines, and that study included respondents who were physically disabled. However, even with these differences, our estimate was not practically different from that of that study, given their estimate and wide CI (25).

We found no association between physical activity level and mortality in the younger age group. It may be that our follow-up period was not long enough, especially to capture deaths associated with chronic conditions most closely associated with physical activity level. In our cohort, only 4.8% of adults aged 25 to 39 at baseline died during follow-up. One study that examined this association among those aged 20 to 44 years did find an association between physical activity and mortality; however, this study had follow-up data for 42 years (11). Researchers may wish to examine this association in younger age groups with a longer follow-up period and multiple measures of physical activity during the follow-up period.

Our findings for adults aged 70 or older were consistent with those in adults aged 40 to 69. Although the difference in the magnitude of the association of the HRs or the PAF between the 2 groups was not large, the association may have been diluted in those aged 70 or older. These adults may have decreased their physical activity level as they aged (26), so we may have had older adults in our sample who previously were active and became less active. This may have resulted in the measure of physical activity in the older age group being an underestimation of their lifetime activity and thereby a dilution of the association observed for the older age group.

Our study has limitations. First, we used observational data, which may have biased the observed associations by introducing confounding factors. We attempted to reduce such bias by controlling for several factors; however, we were not able to control for all potential confounding factors and may have over-adjusted for others. For example, participation in other unhealthy behaviors, such as poor diet, may be related to physical inactivity (27). Because some variables (eg, hypertension, BMI) may be intermediate variables on the causal pathway between physical activity and mortality, controlling for these variables may have been an over-adjustment. We did not adjust for additional chronic diseases (eg, heart disease, diabetes) because of this concern. Second, NHIS physical activity data are derived from self-reported information, and reporting bias can result in high estimates of physical activity (28). However, individuals overestimating their physical activity would lead to a more conservative estimate of this association. Third, the physical activity measure was based only on leisure-time activity, which may have resulted in an underestimation of physical activity levels if individuals’ work hours were considered. Fourth, only a single baseline assessment was available. A longer follow-up period is desirable to minimize censoring; however, the longer follow-up time also means that there is a longer interval between baseline physical activity assessment and the event. In a previous study, researchers concluded that the relative risk of mortality associated with physical inactivity is underestimated when derived from a prospective study using a single baseline measurement (29). Finally, reverse causation may explain some of our association, because adults may have been ill at the time of the baseline measure, potentially influencing their physical activity level and risk of mortality. This was addressed in 2 ways. First, adults identified as physically disabled or who died in the same quarter of the year as interviewed were excluded from the study. Second, we conducted sensitivity analyses that removed adults who died in the first 2 years of follow up, and results were similar.

Our study also has strengths. First, the prospective cohort design of the study allowed us to better examine causality. Second, information on many covariates was available, which allowed us to adjust our models for confounding factors. Third, our physical activity levels were consistent with current guidelines. Finally, the NHIS is nationally representative and has near complete mortality follow-up for a long period. Because our goal was to estimate the percentage of deaths associated with inadequate physical activity levels in the US population, nationally representative data are preferable, and the near-complete follow-up ensured the generalizability of our study findings.

Levels of physical activity inadequate to meet current guidelines are associated with a significant proportion of deaths in US adults. Increasing adults’ physical activity levels to meet guidelines and *Healthy People 2020* objectives is a way to reduce the risk of premature death in the United States.
